# Exploring a Rare Pulmonary Coinfection: Cryptococcal Pneumonia and Exophiala dermatitidis in an Immunocompetent Host

**DOI:** 10.7759/cureus.61085

**Published:** 2024-05-25

**Authors:** Akshay Kohli, Zeyad J Rifai, Nathalie Foray

**Affiliations:** 1 Pulmonary and Critical Care Medicine, Southern Illinois University School of Medicine, Springfield, USA; 2 Internal Medicine, Southern Illinois University School of Medicine, Springfield, USA

**Keywords:** pneumonia, bronchoalveolar lavage (bal), exophiala dermatitidis, cryptococcus neoformans (c. neoformans), fungal pneumonia

## Abstract

Pulmonary cryptococcosis is becoming increasingly common in immunocompetent hosts, manifesting with variable clinical presentations ranging from asymptomatic colonization to severe pneumonia. Radiological findings are non-specific, such as nodular infiltrates, mass-like lesions, and mediastinal lymphadenopathy. We present a case of a 61-year-old woman with *Cryptococcus neoformans* pneumonia coinfected with *Exophiala dermatitidis*, an unusual occurrence in an immunocompetent host and the first of its kind. This coinfection posed significant diagnostic challenges due to the rare occurrence of each individual organism in immunocompetent patients as well as the difficulty of their laboratory diagnosis. Treatment regimens, particularly in coinfections, warrant careful consideration to mitigate mortality risk. This case underscores the importance of comprehensive diagnostic strategies and optimized treatment regimens for rare fungal coinfections in immunocompetent hosts.

## Introduction

Cryptococcus is a fungal infection caused by *Cryptococcus neoformans* or *Cryptococcus gattii*. It primarily affects individuals with weakened immune systems, such as those with HIV/AIDS, organ transplant recipients, or those undergoing immunosuppressive therapy [1-3]. In the immunocompromised individual, cryptococcal pneumonia can present as a disseminated infection with an interstitial lung pattern and with or without lymphadenopathy [4]. However, in an immunocompetent host, pulmonary cryptococcosis tends to be indolent. Radiographic findings commonly include single or multiple nodules or mass-like lesions or consolidation. Cavitary lesions, pleural effusion, or mediastinal lymphadenopathy are rare in immunocompetent patients [5,6].

*Exophiala dermatitidis* also known as *Wangiella dermatitidis* is a black fungus, found mainly in soil and dead plant material, in areas of high humidity such as steam baths, bathrooms, and dishwashers [7-9]. It is a known cause of skin and subcutaneous tissue infections in immunocompromised and organ transplant recipients [7,10]. Pneumonia caused by *E. dermatitidis* is exceedingly rare and most commonly, it is isolated from respiratory samples of cystic fibrosis (CF) patients [11,12]. Rarely, it has also been reported in non-CF patients with bronchiectasis [13,14].

We describe an interesting case of a patient with Cryptococcal neoformans pneumonia coinfected with *E. dermatitidis*.

## Case presentation

**Figure 1:**
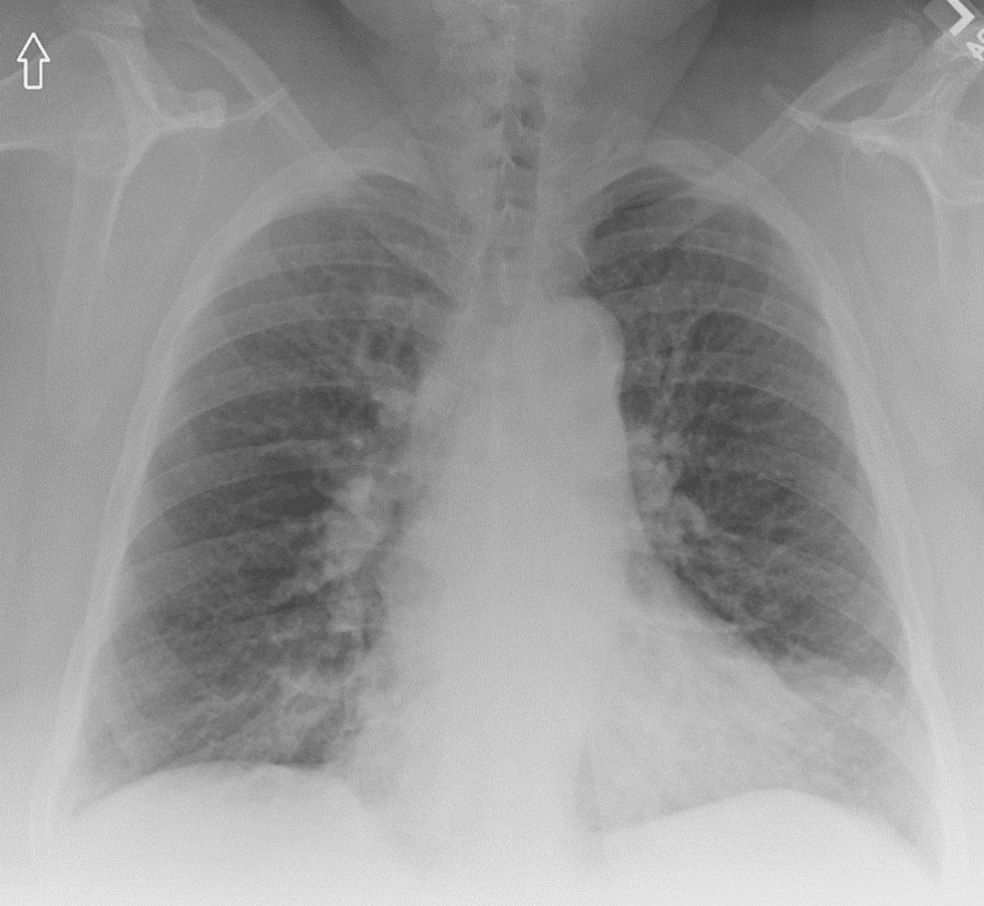
Chest radiograph on admission showing bilateral diffuse interstitial opacities.

A 61-year-old woman with a medical history of type II diabetes, hypothyroidism, chronic obstructive pulmonary disorder (COPD), obstructive sleep apnea on positive airway pressure therapy, factor V Leiden deficiency on rivaroxaban presented to the emergency department with symptoms including shortness of breath, fever, chills, and malaise on and off for about a month. A chest X-ray revealed evidence of bilateral interstitial infiltrates (Figure 1).

**Figure 2:**
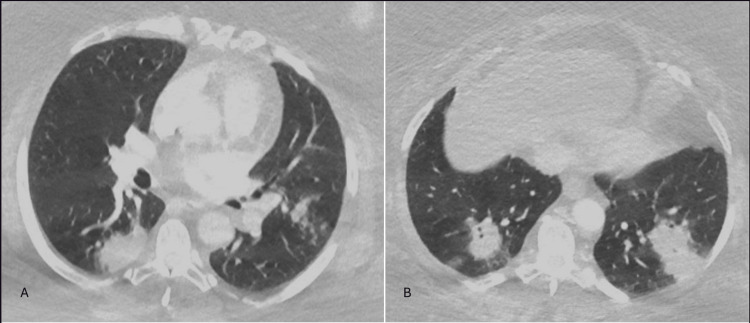
Admission chest CT scan (lung window) showing bilateral, scattered nodular opacities. Panel A showing an axial view with right lower lobe opacities. Panel B showing bilateral lower lobe dense nodular opacities.

She remained on room air and a complete blood count did not reveal leukocytosis; thus, she was subsequently discharged home with a prescription of amoxicillin-clavulanic acid and doxycycline to treat community-acquired pneumonia. However, the patient returned the following evening with worsening symptoms and computed tomography (CT) of the chest revealed bilateral consolidative opacities in the lower lobes suggestive of pneumonia (Figure 2).

During this hospitalization, the patient’s infectious work-up, including sputum culture and gram stain, atypical pneumonia workup including *Mycoplasma pneumonia *immunoglobulin M antibody, *Streptococcus pneumoniae* urine antigen, and urine Legionella antigen were negative. The patient remained afebrile and her shortness of breath improved following treatment for community-acquired pneumonia via three days of ceftriaxone and azithromycin during her admission and two days of cefpodoxime upon discharge.

**Figure 3:**
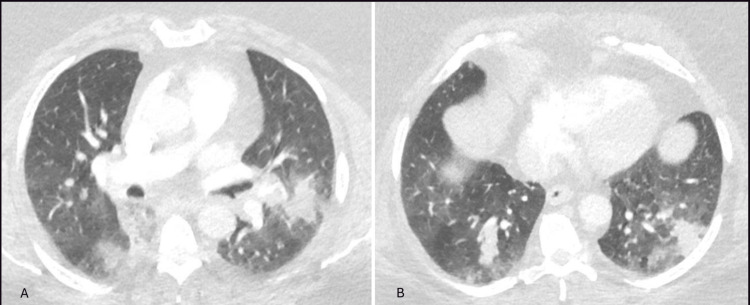
Axial view of chest CT scan (lung window) showing worsening bilateral opacities (as compared to the CT scan on admission). Panel A showing new left lower lobe dense opacities. Panel B showing persistent bilateral lower lobe opacities.

The patient returned to the hospital a few days later with shortness of breath and diffuse wheezing. She was afebrile and denied sputum production, and CT of the chest revealed worsening of the bilateral opacities, now appearing as multifocal bilateral dense nodular opacities (Figure 3).

**Figure 4:**
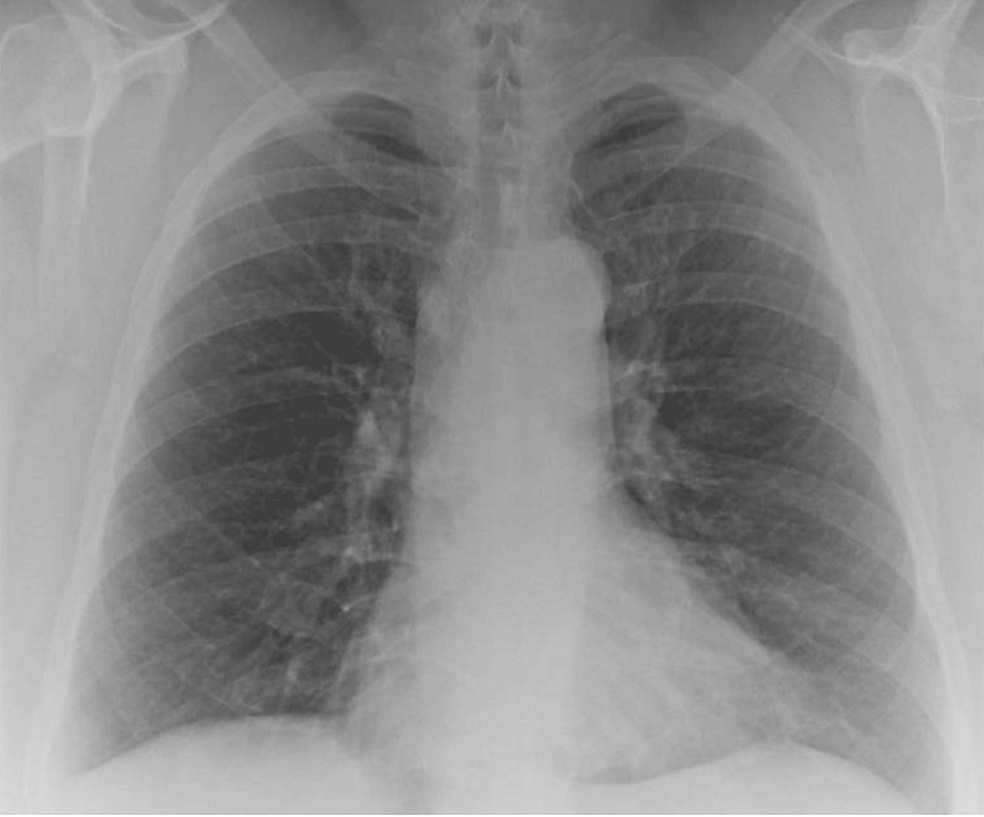
Post-treatment chest radiograph showing significant improvement of bilateral diffuse interstitial opacities.

She was subsequently treated with a five-day course of corticosteroids for a COPD exacerbation and was initiated on piperacillin-tazobactam for suspected nosocomial pneumonia. Given the negative infectious workup, and worsening pulmonary opacities on imaging despite multiple courses of antibiotics, we decided to pursue bronchoscopy with bronchoalveolar lavage (BAL). Notable, non-obstructing, green, tenacious, thick secretions were found in the right mainstem bronchus, the bronchus intermedius, the right upper lobe, the right middle lobe, and the right lower lobe bronchi. Notable, non-obstructing, green, mucoid, tenacious, thick secretions were also found in the left upper lobe. There were no endobronchial masses obstructing the airways. A BAL of the lingula was completed. Initial gram stain and cultures were negative for bacterial growth; however, fungal culture from the BAL revealed *C. neoformans* and *E. dermatitidis*. This raised the concern for an immunocompromised status and potential disseminated disease, therefore a human immunodeficiency virus (HIV) testing, quantiferon (for tuberculosis), a lumbar puncture, and serum Cryptococcal antigen were obtained with negative findings. The remainder of her fungal immunodiffusion testing, including aspergillosis, blastomycosis, coccidioidomycosis, histoplasmosis immunodiffusion, and serum immunoglobulin levels were unremarkable. The patient was diagnosed with coinfection of *C. neoformans* pneumonia and *E. dermatitidis* and was started on a six-month regimen of Fluconazole. Our patient maintained close, multidisciplinary outpatient follow-up with gradual resolution of her symptoms. Upon completing her antifungal regimen, the patient was asymptomatic and serum fungal antigens remained negative with significant improvement in radiographic findings (Figure 4).

## Discussion

Cryptococcosis is a fungal infection caused by *C. neoformans* and *C. gattii*. *C. neoformans* can infect both immunocompromised and apparently immunocompetent patients. *C. gattii*, on the other hand, has long been thought to be a pathogen of immunocompetent persons [15]. Among the non-HIV patients with cryptococcal pneumonia, severe diabetes mellitus with organ damage is a major risk factor [16,17]. The clinical presentation of pulmonary cryptococcosis is variable and depends on the immune status of the patient. As noted above, it can present with non-specific symptoms such as cough, dyspnea, chest pain, and fever. Approximately half of the immunocompetent patients with pulmonary cryptococcosis are asymptomatic. Thus, diagnosis is usually delayed.

Diagnosis of pulmonary cryptococcosis is usually based on a combination of clinical, radiological, and laboratory findings. Culture, direct microscopy, histopathology, serological markers, or molecular detection are used to confirm the diagnosis [18]. Identification of positive culture from BAL in a patient with appropriate clinical symptoms and radiological findings is key to diagnosis. Antigens test from serum or blood can be negative unless the infection is disseminated. Once pulmonary cryptococcosis is diagnosed or suspected, at-risk patients should undergo lumbar puncture to test cerebrospinal fluid for cryptococcal antigen, because the presence of CNS disease alters the therapy [19]. Therapy for isolated pulmonary cryptococcosis depends on the immune status of the patient and the severity of the disease. Fluconazole 400 mg per day for 6-12 months is the first-line therapy for mild-moderate disease. Severe disease requires more intensive therapy with amphotericin B and flucytosine for induction therapy and then fluconazole for consolidation therapy [19]. *E. dermatitidis* has become an increasingly common pathogen isolated from the respiratory secretions of CF patients. It has a wide spectrum of manifestations ranging from mere colonization to invasive and potentially fatal infection [11-13,20]. Recent data and literature review suggest that most of the cases that manifested as invasive diseases had predisposing factors such as peritoneal dialysis, corticosteroid use, HIV infection, cancer, or diabetes mellitus [21,22]. In another case report, a patient with non-CF bronchiectasis cultured nontuberculous mycobacteria (NTM) along with *E. dermatitidis* from the respiratory sample. In that case, pneumonia and pulmonary manifestations were deemed to be caused by NTM, and *E. dermatitidis* was considered a colonizer [14].

As it is a rarely encountered species, the incidence and prevalence of *E. dermatitidis* infection in the general population are unknown, particularly in visceral involvement such as the lungs. Pulmonary *E. dermatitidis* infection has been discussed in CF and non-CF bronchiectasis patients. In a study by Lebecque et al., *E. dermatitidis* was identified in 9 out of 154 (5.8%) CF patients; these samples were obtained via sputum or deep pharyngeal aspirate following chest physiotherapy [23]. The challenge of isolating *E. dermatitidis* and a prolonged incubation period of about two weeks might be one of the factors leading to low isolation rates. Of note, all culture-positive patients in this study interestingly had pancreatic insufficiency [23].

There are no guidelines for appropriate treatment of *E. dermatitidis* pulmonary infections. Previous case reports have shown good outcomes with monotherapy or combination therapy with amphotericin B, 5-fluorocytosine, itraconazole, and other antifungals [13,22,24-26]. Additionally, there is no standardized duration of therapy for either immunocompetent or immunocompromised hosts, resulting in extended treatment regimens. Previous reports have suggested voriconazole, itraconazole, and amphotericin B to be the most potent, as evidenced by the minimum inhibitory concentration [22,24,26,27]. BAL is a crucial component in the diagnosis of fungal pneumonia, particularly in immunocompetent patients. This is especially true in cases when it is hard to delineate a particular organism just by history and clinical presentation. The strength of BAL lies in its capacity to directly access the lower respiratory tract, providing a representative specimen amenable to comprehensive microbiological, cytological, and histopathological evaluation. In the workup of recurrent pneumonia or pneumonia not responding to antibiotics, BAL plays an important role in potentially identifying the causative organism [28]. In our patient, BAL fluid cultures played a pivotal role in diagnosing both *C. neoformans* and *E. dermatitidis* infection.

Our patient was coinfected with Cryptococcal pneumonia necessitating further consideration and discussions about whether the symptoms were due to Cryptococcus pneumonia alone and whether *E. dermatitidis* was just a colonizer. After careful consideration and multidisciplinary team discussion, we elected to treat our patient with oral antifungal therapy for cryptococcal infection (duration of therapy catered toward cryptococcal pneumonia). Our case highlights the presence of two exceedingly rare fungal organisms coinfecting an immunocompetent host.

## Conclusions

The occurrence of coinfection involving *C. neoformans* and *E. dermatitidis* in an immunocompetent individual raises several questions regarding the pathogenesis and clinical management of fungal pneumonia. Physicians should maintain a high degree of suspicion and promptly utilize diagnostic tests such as bronchoscopy with BAL when faced with unresolved or recurrent pneumonia, even in patients with apparently preserved immunological function. Combining BAL with other diagnostic modalities such as serum biomarkers and molecular techniques can improve the overall diagnostic accuracy for invasive fungal infections. Last, the clinical relevance of the isolation of *E. dermatitidis* in patients is still unknown. Active surveillance might be necessary for such patients should symptoms persist or recur.
